# Supplementary material for: GWAS for quantitative resistance phenotypes in Mycobacterium tuberculosis reveals resistance genes and regulatory regions
Source: Nat Commun. 2019 May 13;10:2128. doi: 10.1038/s41467-019-10110-6 (PMC6513847; doi:10.1038/s41467-019-10110-6)
Supplement: Supplementary file 1 — Supplementary Information [file 41467_2019_10110_MOESM1_ESM.docx]

GWAS for quantitative resistance phenotypes in *Mycobacterium tuberculosis* reveals resistance genes and regulatory regions

Farhat et al.

Supplementary Information

**Supplementary Figure 1: MIC histograms for 12 drugs.** Dotted red line represents the WHO recommended critical concentration on 7H10 media, and the blue line represents the lower limit of Cmax from published pharmacokinetic studies (Supplementary Table 2, and supplementary reference 1).

**Supplementary** **Figure 2:** **QQ-plots of GWAS results to 12 drugs without (top) and with population structure control (below) for each drug.** Panels correspond to the drugs as follows: A:INH, B:RIF, C:RBU, D:EMB, E:CAP, F:AMI, G:KAN, H:ETA, I:PZA, J:STR, K:MXF, L:LIN (drug abbreviations in Supplementary Table 2).

**Supplementary Figure 3: Variant distribution in the 13 validated non-canonical loci, *mymA* (candidate that failed validation) and three known resistance genes for comparison.** Red lines indicate variants predicted to have a high impact in coding genes i.e. were either nonsense SNSs and frameshift indels. All other variants were missense variants or intergenic variants (no silent variants were included in the burden score). Predicted transcription start sites are shown in dashed blue vertical lines for intergenic regions (from Shell et al PMID 26536359). *pncA* gene mutations are known to be causative of PZA resistance, similarly *katG* mutations can cause INH
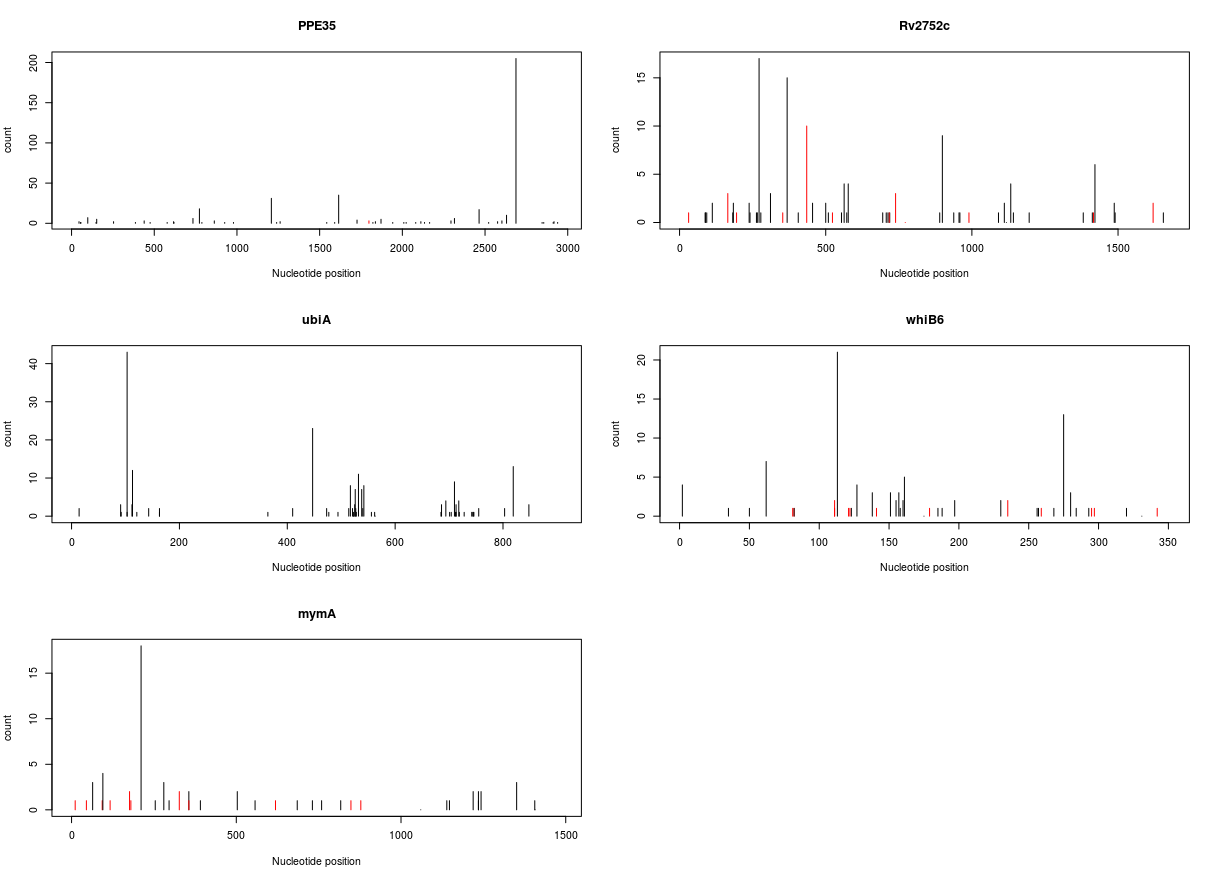
resistance and *rpoB* mutations RIF resistance.


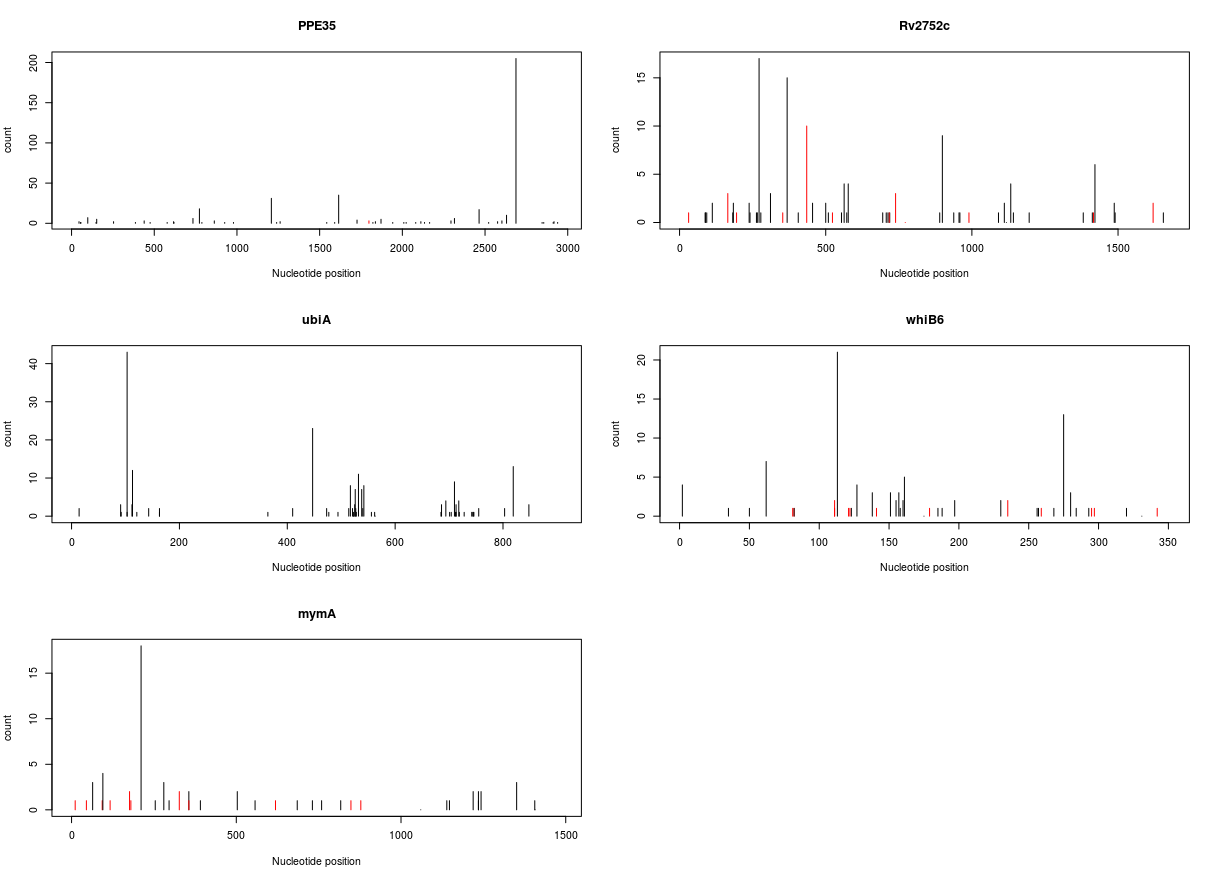
**
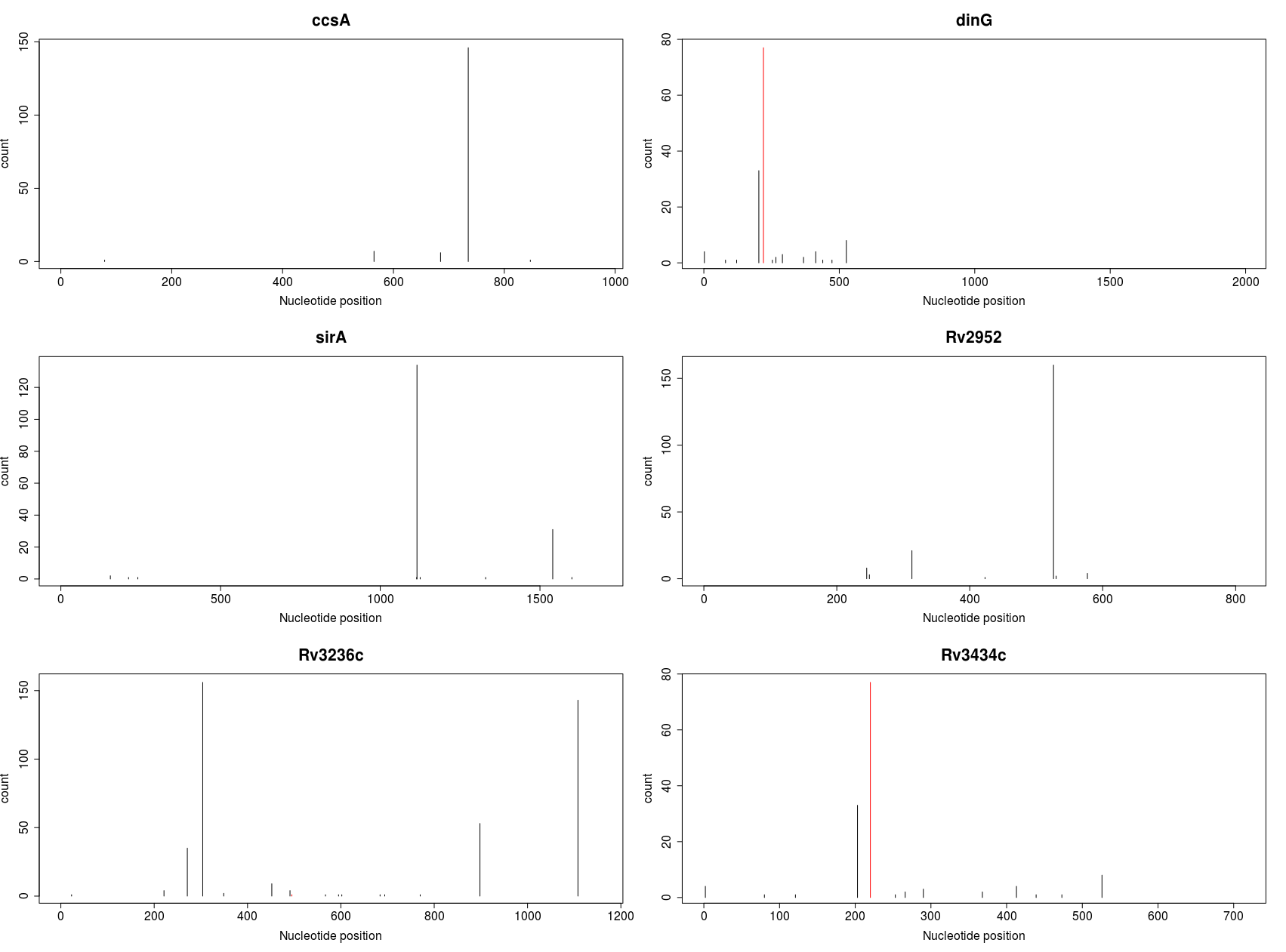
**­­


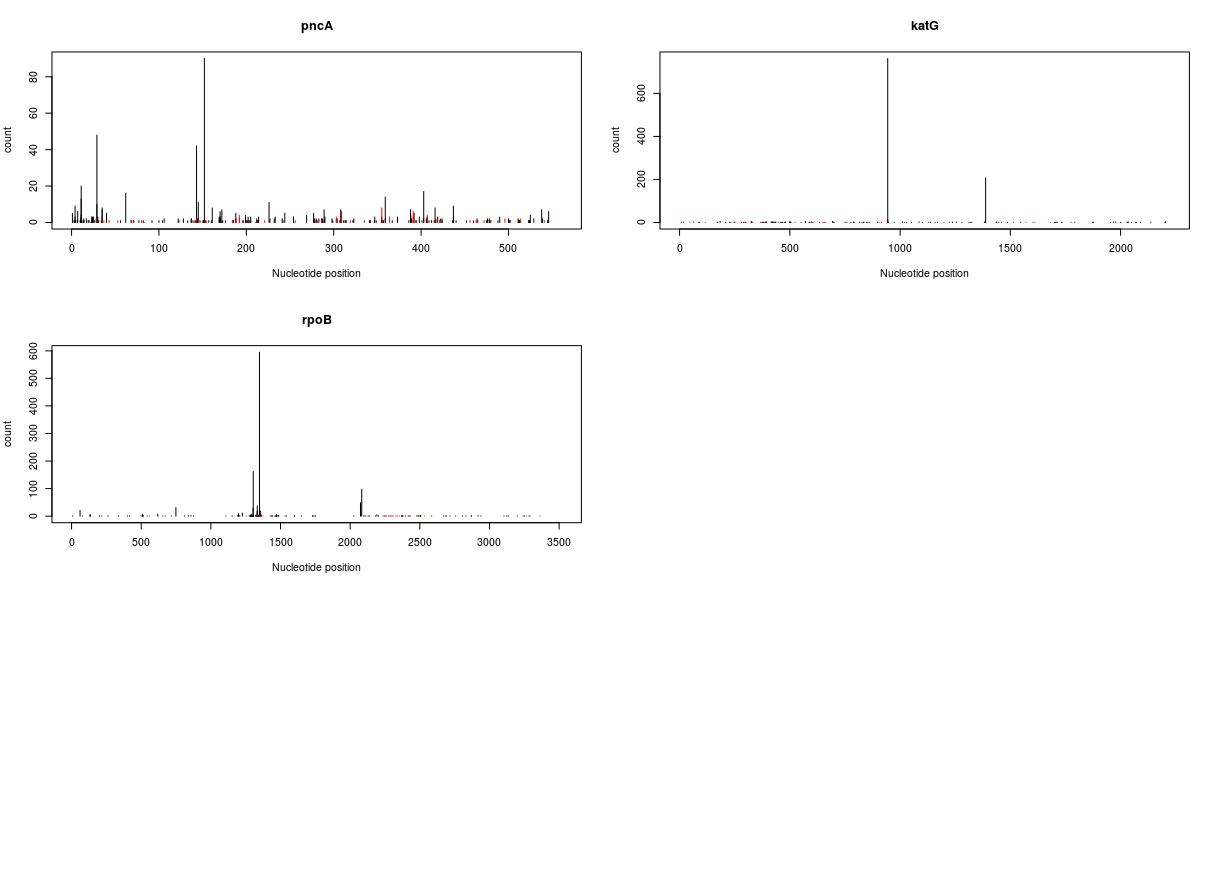

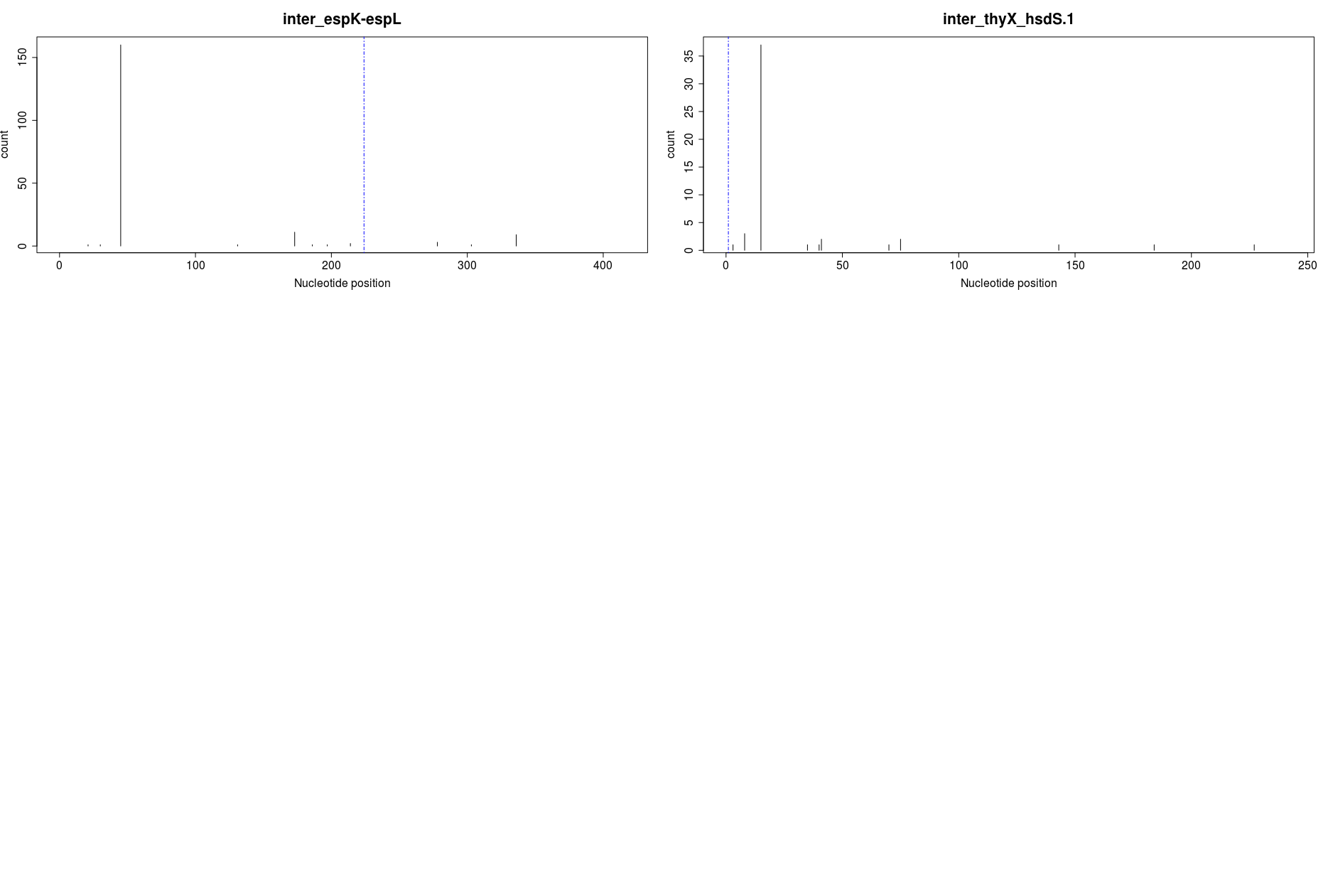

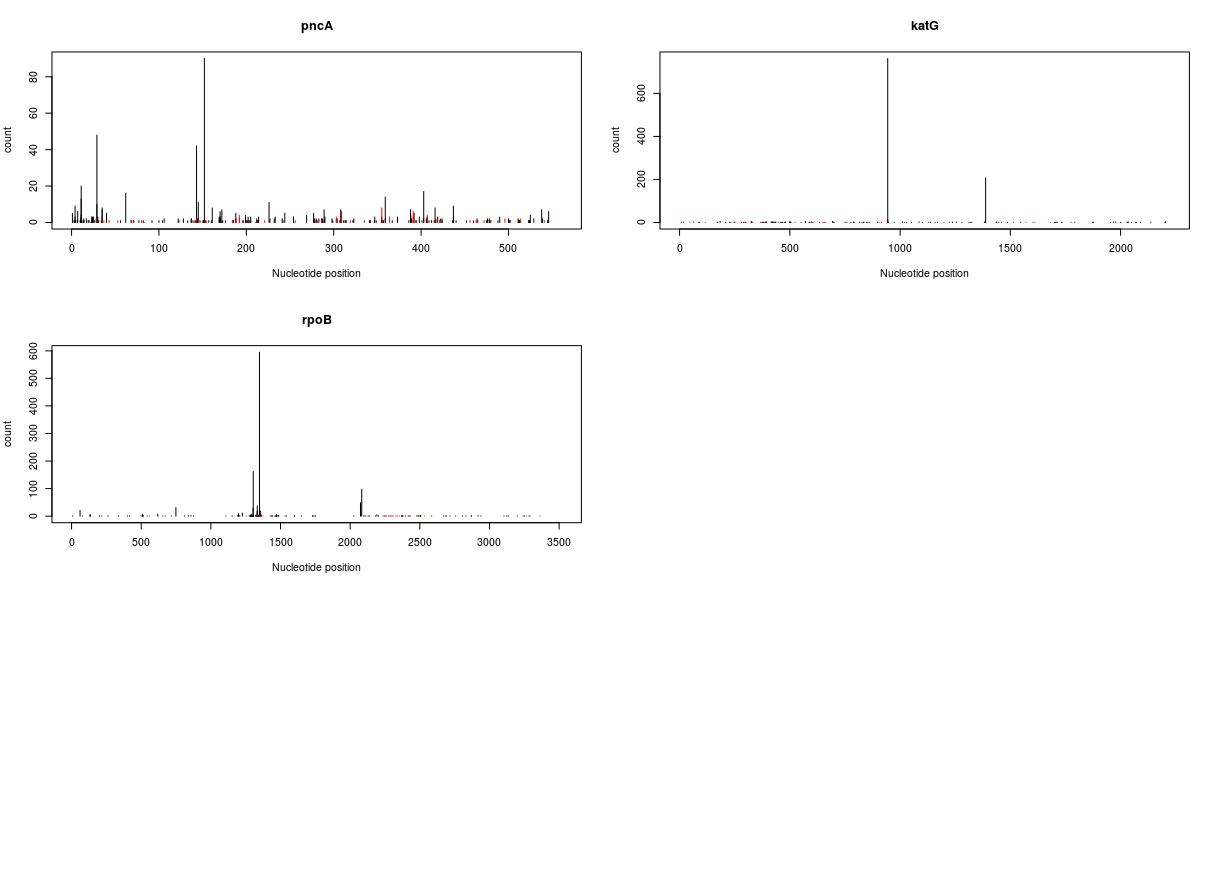


**Supplementary Figure 4: Neighbor joining tree of 1452 isolates built using MEGA-5 (PMID 21546353) and visualized using iTOL (PMID 27095192).** Red indicates lineage 4, purple lineage 3, blue lineage 2, and green lineage 1.

**
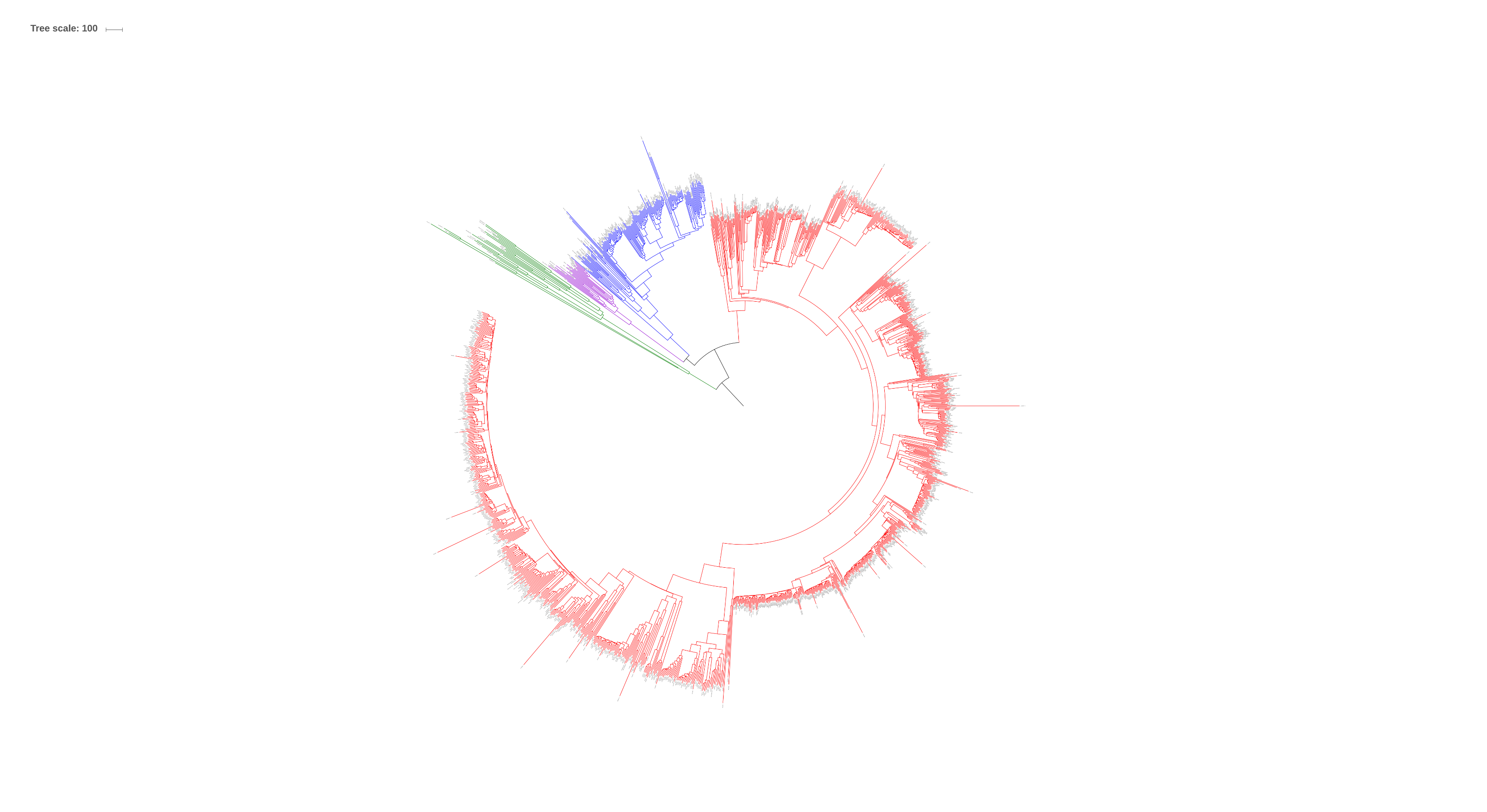

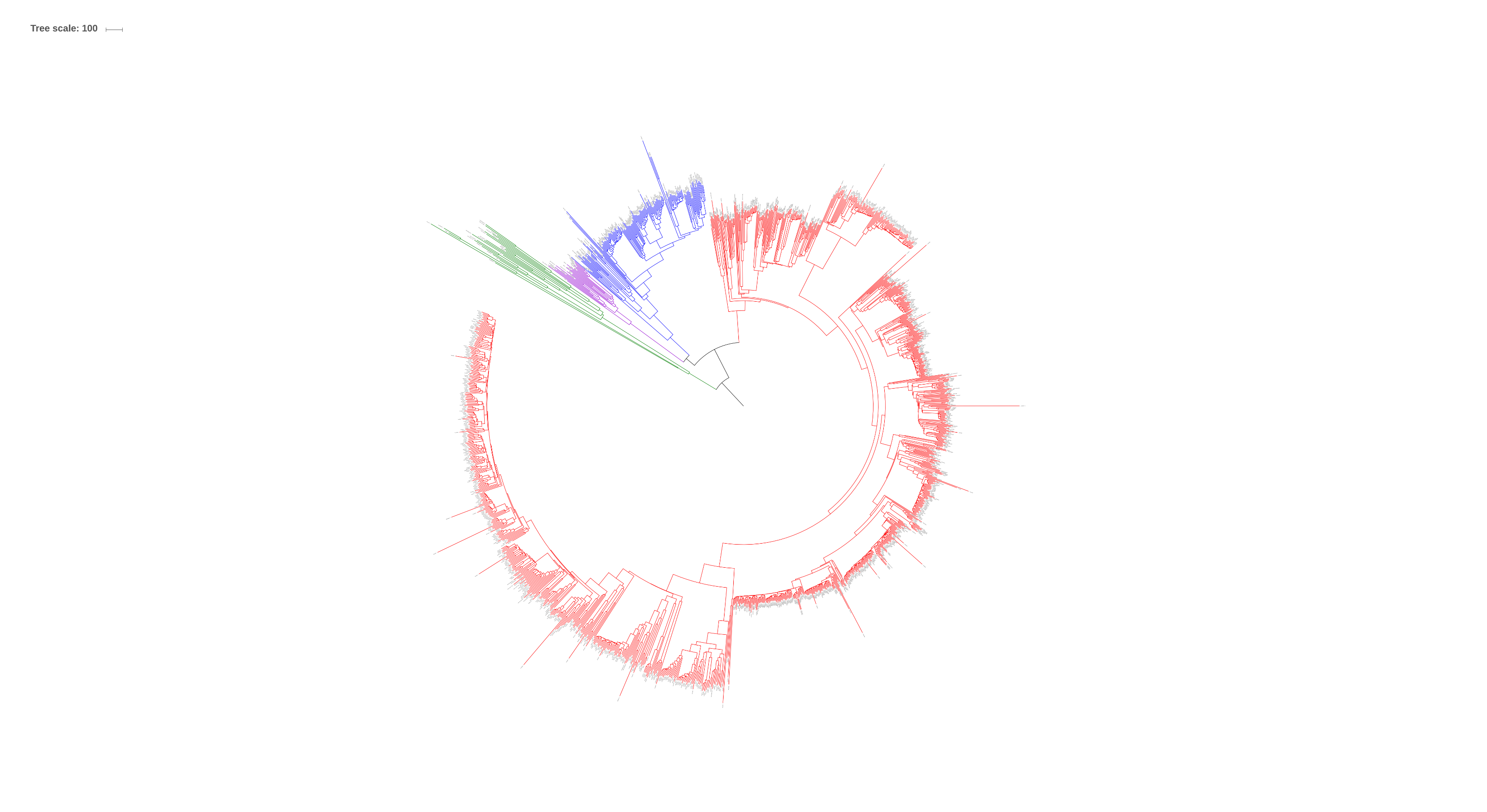
**

**Supplementary Table 1: Promoter and gene body variants and their lineage distribution**

|  |  |  | |  | |  | |  | |
| --- | --- | --- | --- | --- | --- | --- | --- | --- | --- |
|  |  | **embAB promoter variants (any of the below)** | | | | | **embB coding variant** | |  |
| Lineage | Total count | C16T promoter embB | C16G promoter embB | | C16A promoter embB | | M306V embB | |  |
| 1.1.1 | 3 | 0 | 0 | | 0 | | 0 | |  |
| 1.1.2 | 5 | 0 | 0 | | 0 | | 0 | |  |
| 1.1.3 | 2 | 0 | 0 | | 0 | | 0 | |  |
| 1.2.1 | 5 | 0 | 0 | | 0 | | 1 | |  |
| 1.2.2 | 4 | 0 | 0 | | 0 | | 1 | |  |
| 2 | 9 | 0 | 0 | | 0 | | 0 | |  |
| 2.1 | 1 | 0 | 0 | | 0 | | 0 | |  |
| 2.2 | 24 | 0 | 0 | | 0 | | 8 | |  |
| 2.2. | 140 | 1 | 2 | | 1 | | 41 | |  |
| 3 | 23 | 0 | 0 | | 0 | | 2 | |  |
| 4 | 171 | 5 | 4 | | 0 | | 12 | |  |
| 4.1 | 447 | 4 | 2 | | 3 | | 26 | |  |
| 4.2.1 | 6 | 0 | 0 | | 0 | | 2 | |  |
| 4.2.2 | 6 | 1 | 0 | | 0 | | 1 | |  |
| 4.3 | 584 | 2 | 9 | | 5 | | 87 | |  |
| 4.5 | 2 | 0 | 0 | | 0 | | 0 | |  |
| 4.6.1 | 21 | 0 | 0 | | 0 | | 13 | |  |
| 4.7 | 11 | 0 | 0 | | 0 | | 0 | |  |
| 4.8 | 20 | 0 | 0 | | 0 | | 1 | |  |
| 4.9 | 1 | 0 | 0 | | 0 | | 0 | |  |
| 5 | 1 | 0 | 0 | | 0 | | 0 | |  |
| BOV | 3 | 0 | 0 | | 0 | | 2 | |  |

|  |  | **inhA promoter variant** | **inhA codon 21 variants** | | |
| --- | --- | --- | --- | --- | --- |
| Lineage | Total count | C15T promoter inhA | I21M inhA | I21V inhA | I21T inhA |
| 1.1.1 | 3 | 0 | 0 | 0 | 0 |
| 1.1.2 | 5 | 0 | 0 | 0 | 0 |
| 1.1.3 | 2 | 0 | 0 | 0 | 0 |
| 1.2.1 | 5 | 3 | 0 | 0 | 0 |
| 1.2.2 | 4 | 0 | 0 | 0 | 0 |
| 2 | 9 | 0 | 0 | 0 | 0 |
| 2.1 | 1 | 0 | 0 | 0 | 0 |
| 2.2 | 24 | 3 | 0 | 0 | 0 |
| 2.2. | 140 | 16 | 0 | 2 | 0 |
| 3 | 23 | 3 | 0 | 0 | 0 |
| 4 | 171 | 8 | 0 | 0 | 0 |
| 4.1 | 447 | 62 | 0 | 1 | 3 |
| 4.2.1 | 6 | 3 | 0 | 0 | 0 |
| 4.2.2 | 6 | 0 | 0 | 0 | 0 |
| 4.3 | 584 | 116 | 1 | 1 | 35 |
| 4.5 | 2 | 0 | 0 | 0 | 0 |
| 4.6.1 | 21 | 0 | 0 | 0 | 0 |
| 4.7 | 11 | 2 | 0 | 0 | 0 |
| 4.8 | 20 | 4 | 0 | 0 | 0 |
| 4.9 | 1 | 0 | 0 | 0 | 0 |
| 5 | 1 | 0 | 0 | 0 | 0 |
| BOV | 3 | 2 | 0 | 0 | 0 |

**Supplementary Table 2: Summary of antibiotic concentrations tested to determine MIC at National Jewish Health (NJH) Mycobacterial Laboratory.** Note critical concentrations are based on the WHO 2014 recommendations. *INH: isoniazid, RIF: rifampicin, EMB: ethambutol, PZA: pyrazinamide, STR: streptomycin, ETA: ethionamide, CIP: ciprofloxacin, LEVO: levofloxacin, OFX: ofloxacin, MXF: moxifloxacin, AMI: amikacin, KAN: kanamycin, CAP: capreomycin, PAS: para-aminosalicylic acid, CYS: cycloserine. LIN: linezolid. All testing was performed on Middlebrook 7H10 with the exception of **PZA which was tested in MGIT 960 radiometric system.

| Drug* | Critical Concentration (CC) (mg per L) | Peak Serum Conc (mg per L)[ref^50^] | Low concentrations (mg per L) < CC | | High concentrations (mg per L) > CC | | | | | |
| --- | --- | --- | --- | --- | --- | --- | --- | --- | --- | --- |
|  |  |  | Conc1 | Conc2 | Conc1 | Conc2 | Conc3 | Conc4 | Conc5 | Conc6 |
| INH | 0.2 | 3-5 | 0.05 | 0.1 | 0.4 | 0.6 | 1.0 | 2.0 | 4.0 | 5.0 |
| RIF | 1.0 | 8-24 | 0.25 | 0.5 | 2.0 | 3.0 | 5.0 | 8.0 | 10.0 | 50 |
| RBU | 0.5 | 0.3-0.9 | 0.125 | 0.25 | 0.6 | 0.75 | 0.875 | 1.0 | 1.5 | 2.5 |
| EMB | 5.0 | 2-5 | 1.25 | 2.5 | 6.0 | 7.0 | 8.0 | 9.0 | 10.0 | 15.0 |
| PZA** | 100 | 30-50 | 25 | 50 | 120 | 150 | 175 | 200 | 300 | 500 |
| STR | 2.0 | 25-50 | 0.5 | 1.0 | 4.0 | 6.0 | 10.0 | 16.0 | 20.0 | 100 |
| MXF | 0.5 | 2-10 | 0.125 | 0. 25 | 1.0 | 2.0 | 4.0 | 6.0 | 8.0 | 10.0 |
| AMI | 5.0 | 3-5 | 1.25 | 2.5 | 6.0 | 10.0 | 15.0 | 30.0 | 50.0 | 100 |
| KAN | 5.0 | 35-45 | 1.25 | 2.5 | 6.0 | 10.0 | 15.0 | 30.0 | 50.0 | 100 |
| CAP | 4.0 | 35-45 | 1.0 | 2.0 | 5.0 | 10.0 | 15.0 | 30.0 | 50.0 | 100 |
| ETA | 5.0 | 35-45 | 1.25 | 2.5 | 6.0 | 8.0 | 10.0 | 15.0 | 20.0 | 50 |
| LIN | 1.0 | Mean 4.9 | 0.25 | 0.5 | 2.0 | 3.0 | 4.0 | 6.0 | 8.0 | 10.0 |

**Supplementary Table 3:** **Culture, MIC and DST methodology for other labs than at NJH.**

* Drug abbreviations expanded in Supplementary Table 2 ** FLQ drugs tested included CIP, LEVO, OFX, MXF, and GATI.

| Lab | Culture method and media | Colony purification | Test | Drug Sensitivity Testing* |
| --- | --- | --- | --- | --- |
| Mass State Laboratory (MSLI) Boston, USA | Radiometric BACTEC 460 TB system (Becton-Dickinson) | No | MIC | Middlebrook 7H10 agar plates with the following concentrations (mg per L) :  1- INH 0.2, 1, and 5  2- RIF 1  3- EMB 5  4- STR 2, 10  5- CS 3.75, 7.5, 15, 30, 45, 60, 90, 120  6- PAS 0.25, 0.5, 1, 2, 3, 4, 6, 8  7- ETA 1, 2, 3, 4, 5, 7, 10, 14  8- KAN 1, 2, 3, 4, 5, 7, 10, 14  9- CAP 1, 2, 4, 8, 10, 16, 20, 32  10- OFX 0.25, 0.5, 1, 1.5, 2.0, 3, 4, 6, 8, 10  11- MXF 0.125, 0.25, 0.5, 1, 4, 8 |
| RVIM, Bilthoven, Netherlands | MGIT and Middlebrook 7H10 solid | No | MIC | Middlebrooke 7H10 agar dilution^68^ concentrations (mg per L):  1- INH 0.5,1,2,3,5,10,20  2- RIF 1,2,5  3- RBU 2  4- EMB 1,2,5,10,20  5- STR 1,2,5,10,20  6- ETA (PRO) 1,2,3,4,5,,7,10,14  7- AMI 1,2,5,10,20  8- MXF 0.125,0.5,2  9- KAN 1,2,5,10,20  10- CAP 5, 20  11- PZA 10,20,50,100 |
| Institute for tropical medicine (ITM), Antwerp, Belgium | Dubos Agar then Lowenstein-Jensen slant culture | Yes | MIC | LJ medium for the following drugs with the following concentrations (mg per L):  1- INH CC 0.2 (0.05, 0.2, 0.8, 1.6, 3.2)  2- RIF CC 40 (10,20,30,40,80,120)  3- STR CC 4 (1,2,4,8,16)  4- EMB CC 2 (1,2,4,8)  Middlebrook 7H11 for the following drugs and concentrations (mg per L)  1- OFX CC 2 (1-16 with increments of 1)  2- KAN CC 6  3- CAP CC 10  4- ETA CC 10 |
| Socios en Salud (SES), Lima, Peru | Lowenstein-Jensen slant culture | No | DST | Indirect proportions method on LJ for the following drugs and concentrations (mg per L)  1- INH 0.2, 1  2- RIF 40  3- STR 4  4- EMB 2  MIddlebrook 7H11 for the following drugs and concentrations (mg per L)  1- CIP CC 2  2- KAN CC 6  3- CAP CC 10  4- ETA CC 10  5- PAS CC 8  6- CYS CC 30 |

Supplementary References

1. Alsultan, A. & Peloquin, C. A. Therapeutic Drug Monitoring in the Treatment of Tuberculosis: An Update. *Drugs* **74**, 839–854 (2014).
