# Supplementary material for: GWAS for quantitative resistance phenotypes in Mycobacterium tuberculosis reveals resistance genes and regulatory regions
Source: Nat Commun. 2019 May 13;10:2128. doi: 10.1038/s41467-019-10110-6 (PMC6513847; doi:10.1038/s41467-019-10110-6)
Supplement: Supplementary file 3 — Description of Additional Supplementary Files [file 41467_2019_10110_MOESM3_ESM.pdf]

Description of Additional Supplementary Files

Supplementary Data 1– **Isolate description, accession numbers and resistance testing results. (Excel)**

Supplementary Data 2– **Detailed GWAS and validation results at the locus level. (Excel)**

Supplementary Data 3 – **Detailed GWAS and validation results at the site level. (Excel)**

Supplementary Data 4 – **Validation data accession numbers, sources and drug resistance profiles. (Excel)**

Supplementary Data 5 – **Description of genetic variants found in validated loci and their lineage distribution. (Excel)**

Supplementary Data 6 – **Homoplasy (TreeWAS) Results. (Excel)**

Supplementary Data 7 – **Comparison of GWAS hits with published TB drug resistance GWAS hits (Excel)**

Supplementary Data 8 –**R code to reproduce figures and results, contains annotations indicating input files need and code for each figure and table (R code)**
